# Multifrequency Excitation Method for Rapid and Accurate Dynamic Test of Micromachined Gyroscope Chips

**DOI:** 10.3390/s141019507

**Published:** 2014-10-17

**Authors:** Yan Deng, Bin Zhou, Chao Xing, Rong Zhang

**Affiliations:** State Key Laboratory of Precision Measurement Technology and Instruments, Department of Precision Instrument, Beijing 100084, China; E-Mails: zhoub@mail.tsinghua.edu.cn (B.Z.); xc04@mails.tsinghua.edu.cn (C.X.); rongzh@mail.tsinghua.edu.cn (R.Z.)

**Keywords:** micromachined gyroscope chip, dynamic test, multifrequency excitation

## Abstract

A novel multifrequency excitation (MFE) method is proposed to realize rapid and accurate dynamic testing of micromachined gyroscope chips. Compared with the traditional sweep-frequency excitation (SFE) method, the computational time for testing one chip under four modes at a 1-Hz frequency resolution and 600-Hz bandwidth was dramatically reduced from 10 min to 6 s. A multifrequency signal with an equal amplitude and initial linear-phase-difference distribution was generated to ensure test repeatability and accuracy. The current test system based on LabVIEW using the SFE method was modified to use the MFE method without any hardware changes. The experimental results verified that the MFE method can be an ideal solution for large-scale dynamic testing of gyroscope chips and gyroscopes.

## 1. Introduction

Gyroscope chips are key components in micromachined gyroscopes, a type of angular rate sensor widely used in military, automotive, consumer electronics, and other fields [1–3]. They are usually manufactured by a silicon-etching technique. Because of their small size and the imperfect etching technique, precise control of the dynamic chip parameters is difficult, such as the resonant frequency and quality factor. Thus, we have to perform a dynamic test before gyroscope chips can be packaged into gyroscopes. In large-scale manufacturing of gyroscopes, a high efficiency for the dynamic test is urgently required, either for chips or gyroscopes.

The sweep-frequency excitation (SFE) method is the most commonly used dynamic test method for gyroscopes or gyroscope chips [4–6]. A given sweep range (bandwidth) around the resonant frequency is tested by the stepped frequency point by point to plot the frequency response curve, and the dynamic parameters are then extracted. For each frequency point, a stable sine excitation signal is applied, and a stable response signal is then recorded to achieve a high test accuracy. However, this method is extremely time-consuming and cannot yield satisfactory test efficiency, especially in large-scale tests. Recently, search-and-track strategies for the SFE method have been reported in [7], which shorten the test times for the resonant frequency to 1 s and 5 s. The limitations of these methods are that they can only measure the resonant frequency. On the other hand, the use of transient signal excitation such as a step or impulse signal can shorten the test time to 15–20 s, as reported in [8,9]. However, these methods extract the dynamic parameters from the free-decay responses, which are not as stable as the sine response in the SFE method and thus cannot assure test repeatability and accuracy [8,9].

In the current work, a novel multifrequency excitation (MFE) method is proposed to achieve both high efficiency and accuracy. The multifrequency signal was designed to yield a high output signal-to-noise ratio, and a gyroscope-chip dynamic test system was implemented. Finally, the performance of the MFE method was experimentally verified by comparison with the traditional SFE method.

## 2. Principles of the Micromachined Gyroscope Chip and Its Dynamic Test

**Figure 1.**
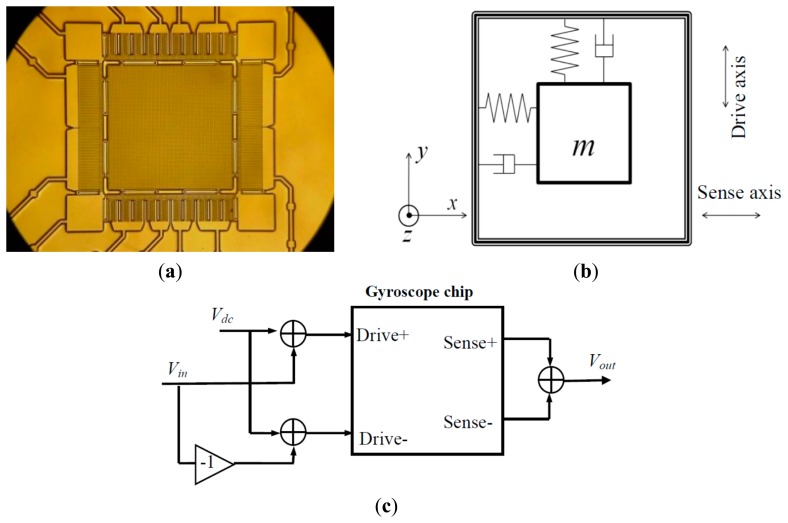
(**a**) Scanning electron microscope (SEM) photograph of a linear vibratory micromachined gyroscope chip; (**b**) Schematic of a linear vibratory micromachined gyroscope chip; (**c**) Schematic of dynamic test of the drive axis input–sense axis output mode.

Figure 1a and b show a scanning electron microscope (SEM) photograph and schematic of a linear vibratory micromachined gyroscope chip. The chip is composed of an orthogonal mass–spring system with a shared sensitive mass and two pairs of electrodes in the two vibratory axes, including the sense axis (*x* axis) and drive axis (*y* axis) [10,11]. First, a sine signal voltage is applied to the drive electrode so that the sensitive mass vibrates along the drive axis. If the gyroscope turns around its sensitive axis (*z* axis) at this instance, the sense electrode will be driven by a Coriolis force and will subsequently vibrate with an amplitude proportional to the angular rate Ω of the sensitive axis. Finally, the vibration is transformed into a capacitance change in the sense electrode pairs and yields a voltage output signal proportional to Ω.

Theoretically, the linear vibratory micromachined gyroscope chip is a second-order vibratory system along the drive and sense axes with the following transfer functions:

(1) $$\begin{array}{l} G_{d}(s)=\frac{Y(s)}{F_{d}(s)}=\frac{1/m}{s^{2}+\omega_{d}/Q_{d}\cdot s+\omega_{d}^{2}}\\ G_{p}(s)=\frac{X(s)}{F_{p}(s)}=\frac{1/m}{s^{2}+\omega_{p}/Q_{p}\cdot s+\omega_{p}^{2}} \end{array}$$

where the subscripts *d* and *p* represent the drive and sense axes, respectively; *X* and *Y* are the displacements of the sensitive mass *m* along the *x* and *y* axes, respectively; *F_p_* and *F_d_* are the forces applied to the mass; *ω_d_* and *ω_p_* are the resonant frequencies; and *Q_d_* and *Q_p_* are the quality factors.

In this study, a dynamic test of the gyroscope chips was conducted on the basis of the principle of electrostatic actuator-capacitance detection, which was described in detail in the authors' previous work [12]. The schematic of the dynamic test of the drive axis input–sense axis output mode is shown in Figure 1c.

In brief, a DC offset *V_dc_* was first added to the excitation signal *V_in_* and its inverted signal –*V_in_*. In the SFE method, *V_in_* = *A*sin(*ω*_0_*t*). Then, the two outputs were applied to a pair of inverted drive electrodes, resulting in inverted electrostatic forces. The summation of the inverted electrostatic force, finally applied to the sensitive mass, has the same frequency *ω*_0_ as *V_in_*. Then, the sensitive mass was driven by the electrostatic force. The relationship between the displacement of the sensitive mass and the electrostatic force can also be expressed as Equation (1). The change in the capacitance of the sense electrode pairs, which has an amplitude proportional to the movement of the sensitive mass, was converted to a voltage and then read out as *V_out_*. Finally, the frequency response can be calculated as

(2) $$H(\omega )=\frac{V_{out}(\omega )}{V_{in}(\omega )}=\frac{F[v_{out}(t)]}{F[v_{in}(t)]}$$

where *V_in_*(*ω*) and *V_out_*(*ω*) are the Fourier transforms of the excitation signal *v_in_*(*t*) and the chip output signal *v_out_*(*t*).

The dynamic parameters such as the resonant frequency, resonant amplitude, and resonant phase were extracted from the frequency response. A single chip needs to be tested under four modes depending on the different input and output electrode (or axis) combinations: drive axis input–sense axis output, sense axis input–drive axis output, sense axis input–sense axis output, and drive axis input–drive axis output.

## 3. Principle and Realization of the MFE Method

### 3.1. Principle of the MFE Method

In the current dynamic test procedure of gyroscope manufacturing, the designed resonant frequency of the chips is approximately 3000 Hz. The quality factor is approximately 200 and the half-power bandwidth is approximately 15 Hz. At the same time, the performance of the second-order vibrational mode's spectrum peak, which is normally at approximately 3200 Hz, is tested, therefore the scanned bandwidth is set as 600 Hz (frequency range: 2700–3300 Hz). A 1-Hz frequency resolution is required. To achieve a high test accuracy, full-period sampling is applied to prevent the picket-fence effect of the fast Fourier transform (FFT)-based spectrum analysis [13]. Furthermore, a response settling time is also considered to exclude the transient process. By adding the 0.2-s full-period sampling and 0.05-s response settling times, the sampling time for one frequency point test is 0.25 s. If the traditional SFE method is used, sine signals with 601 frequencies in the scanned bandwidth are applied individually. Therefore, a 2.5-min sampling time is required to complete a one-mode test, and at least a 10-min sampling time is required to finish a four-mode test of one chip. The test efficiency is obviously very low for a large-scale test.

According to the superposition principle of linear systems [13], if the signal *v_out,k_*(*t*) is the output corresponding to the input signal *v_in,k_*(*t*), *k* = 1, 2……, *N*, the input signal $v_{in,sum}(t)=\sum_{k=1}^{N}v_{in,k}(t)$ will have the output signal of $v_{out,sum}(t)=\sum_{k=1}^{N}v_{out,k}(t)$. Then, the frequency response in Equation (2) could be written as

(3) $$H(\omega )=\frac{V_{out,sum}(\omega )}{V_{in,sum}(\omega )}=\frac{F[v_{out,sum}(t)]}{F[v_{in.sum}(t)]}=\frac{F[\overset{N}{\underset{k=1}{\text{∑}}}v_{out,k}(t)]}{F[\overset{N}{\underset{k=1}{\text{∑}}}v_{in.k}(t)]},k=1,2\ldots \ldots ,N$$

In the MFE method, the 601 sine signals used in the SFE method are added together to generate a multifrequency signal before they are transmitted to the chip. The chip output will be the sum of all the single responses of each sine frequency input. Thus, through input- and output-signal spectrum analysis, the frequency response curve could be plotted using only one excitation and one sampling. To meet the 1-Hz frequency resolution requirement, the shortest full-period sampling time should be 1 s, which is the reciprocal of the frequency resolution. By adding a response settling time of 0.5 s, completing a one-mode test takes 1.5 s, and only 6 s is required to complete a one-chip test. Clearly, the test efficiency is dramatically improved through the MFE method presented in this paper.

### 3.2. Design of the Multifrequency Signal

The major challenge for the MFE method is generating an appropriate multifrequency signal. We desire that the input excitation signal possesses a high enough amplitude to yield a high output signal-to-noise ratio for each frequency. However, adding 601 high-amplitude sine signals together would make the total amplitude exceed the limitations of the data acquisition card output or the chip input, which will cause the actual excitation amplitude to be lower than the designed value or result in direct damage of the chip due to a high-voltage input.

The total amplitude of a multifrequency signal is determined by the amplitudes and initial phases of the total sum of the 601 sine signals. Therefore, the amplitude and initial phase distribution should be carefully designed. To simplify the signal control and analysis, the amplitudes of all frequencies were set to be equal. We studied three initial phase distributions, namely, the zero, random, and linear difference phases, as previously described in [14].

(1) If we adopt the zero phase, the initial phase at the *k*th frequency is expressed as
  (4) $$\theta_{k}=0,k=1,2\ldots \ldots ,N$$
  where *N* is the number of frequencies. The corresponding multifrequency signal can be expressed as
  (5) $$x(t)=\overset{N}{\underset{k=1}{\text{∑}}}A\sin(2\pi f_{k}t)$$
  where *A* is the amplitude of each frequency, and *f_k_* is the frequency of the *k*th signal.
(2) If we adopt the random phase, the initial phase at the *k*th frequency is expressed as
  (6) $$\theta_{k}=\text{rand}\cdot 2\pi ,k=1,2\ldots \ldots ,N$$
  where rand is a uniform random number between zero and one. The corresponding multifrequency signal can be expressed as
  (7) $$x(t)=\overset{N}{\underset{k=1}{\text{∑}}}A\sin(2\pi f_{k}t+\text{rand}\cdot 2\pi )$$
(3) If we adopt the linear difference phase, the initial phase at the *k*th frequency is expressed as
  (8) $$\theta_{k}=\overset{k}{\underset{n=1}{\text{∑}}}\frac{2n\pi }{N}=\frac{k(k+1)\pi }{N},k=1,2\ldots \ldots ,N$$

The corresponding multifrequency signal can be expressed as

(9) $$x(t)=\overset{N}{\underset{k=1}{\text{∑}}}A\sin(2\pi f_{k}t+\overset{k}{\underset{n=1}{\text{∑}}}\frac{2n\pi }{N})=\overset{N}{\underset{k=1}{\text{∑}}}A\sin(2\pi f_{k}t+\frac{k(k+1)\pi }{N})$$

**Figure 2.**
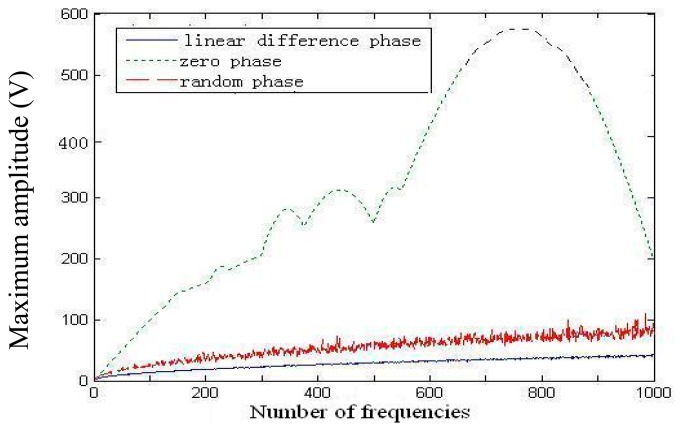
Maximum amplitude of the multifrequency signal *versus* the number of frequencies.

We simulated these three conditions of the initial phase distribution using Matlab. The amplitude of each frequency was 1 V. The first frequency was 2000 Hz, and the frequency interval was 5 Hz. The number of frequencies was set from 1 to 1000. The signal duration time was 0.2 s. Figure 2 shows the maximum amplitude of the multifrequency signal, which varied with the number of frequencies for the three initial phase distributions. The multifrequency signal with the initial linear-difference-phase distribution clearly has the smallest total amplitude. Therefore, in this work, we adopted the initial linear-difference-phase distribution, and the corresponding total amplitude is approximately 30 V for *N* = 601. Typically, the amplitude of the sweep sine signal for a gyroscope chip test is 2 V; thus, the amplitude of each frequency in the multifrequency signal was set to 1/30 of 2 V or 0.065 V.

### 3.3. Test System for Realizing the MFE Method

Virtual instrument technology based on LabVIEW (Instrumentation software released by National Instruments, Austin, TX, USA) helps us realize a test system that is more efficient than traditional instruments and has been adopted for the dynamic testing of gyroscopes in several reports [2,5,8]. In the current study, the multifrequency signal was numerically programmed in LabVIEW and then transmitted to the chip through the NI 4461 data acquisition card, which has two output channels with a 24-bit output resolution and 204.8-kS/s maximum sampling rate. The excitation and response signals were synchronously input into the computer through the NI 4461 card whose input channels have a 24-bit input resolution and 118-dB dynamic range. This test system was previously used to carry out a dynamic test of the chips in the traditional SFE method, and its hardware was described in a previous article [12] by the corresponding author of this paper. The test system was modified very conveniently to use the new MFE method by changing only the system software without any hardware modification.

## 4. Results and Discussion

**Figure 3.**
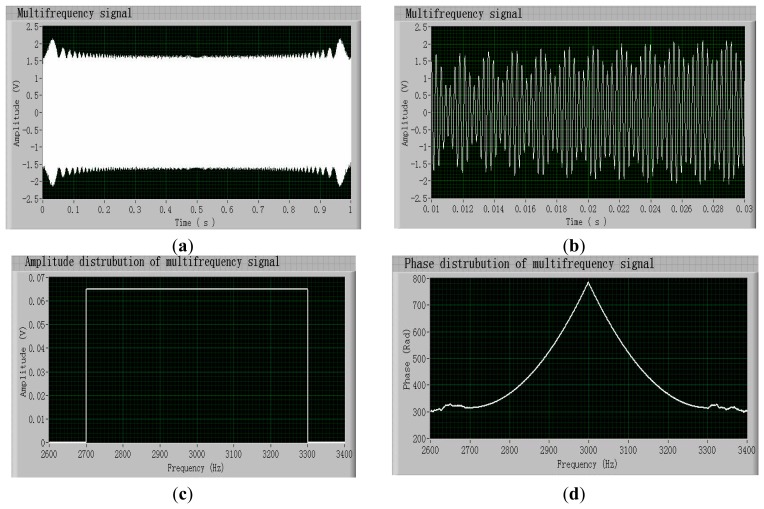
(**a**) Computer-recorded multifrequency signal; (**b**) Partial magnified image of the multifrequency signal; (**c**) Amplitude distribution of the multifrequency signal; (**d**) Phase distribution of the multifrequency signal.

Performance verification of the MFE method and test system was carried out in a clean room where the gyroscope chips were assembled. Figure 3a and b show a computer-recorded multifrequency signal with a 600-Hz bandwidth (frequency range: 2700–3300 Hz) and its partial magnified image. Its corresponding amplitude and phase distributions, which are computed using the FFT-based spectrum analysis [13] without windowing and averaging, are shown in Figure 3c and d. The amplitude in Figure 3c is the peak voltage value of each frequency point. The maximum voltage of the signal is approximately 2 V, as designed.

**Figure 4.**
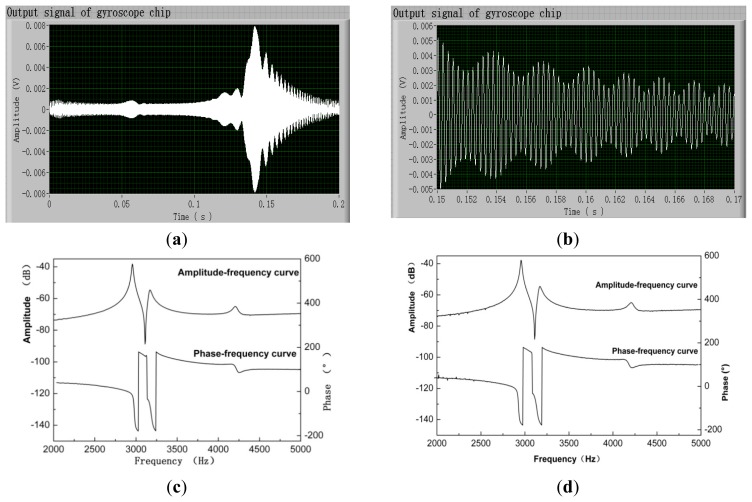
(**a**) Output signal of the gyroscope chip and (**b**) its partial magnified image when using multifrequency excitation (MFE) method. Frequency response of the gyroscope chip tested using the (**c**) sweep-frequency excitation (SFE) method and (**d**) MFE method.

Figure 4 presents the test results of a gyroscope chip with a 5-Hz frequency interval and a wide bandwidth from 2000 Hz to 5000 Hz. The number of frequencies is 601. The excitation signal was applied through its drive axis, and the output signal was read out from its sense axis (the drive axis input–sense axis output mode). When using the MFE method, the input signal is very similar to the waveform shown in Figure 3a. Figure 4a and b show the output signal of the gyroscope chip and its partial magnified image, respectively. In this case, the shortest full-period sampling time was set to be 0.2 s. By adding a response settling time of 0.5 s, completing a one-mode test takes 0.7 s, and only 2.8 s is required to complete a one-chip test. The frequency responses of the gyroscope chip tested using the SFE and MFE methods are almost the same, as shown in Figure 4c and d. The only difference exists in the bandwidth far away from the resonant frequency, where the value is unimportant for the accuracy test. At the frequency points within the bandwidth far away from the resonant frequency, the amplitudes of the output signal are low to approximate 15 μV. The signal-to-noise ratio is very low, resulting in the error in Figure 4c.

The MFE method was applied 10 times to a randomly selected chip and compared with the traditional SFE method. The testing results of the sense axis input–sense axis output and drive axis input–drive axis output modes are listed in Table 1. It is observed that the test repeatability and accuracy for the resonant frequency and the corresponding amplitude and phase can meet the test requirements. The sense axis input–drive axis output and drive axis input–sense axis output modes were tested using the MFE method, and their results are similar to those of the other two modes.

Excitation methods have long been known to be essential for the dynamic tests of mechanical, electronic, and electromechanical systems [15]. The principle of the MFE method is similar to the swept-sine-wave tests using SFE. The swept-sine-wave test continually varies the frequency of a sine wave and applies it to the tested system. In the MFE method, all of these sine waves with continually varying frequencies are first added together and then applied to the tested system at once. This allows for an efficiency improvement. At the same time, every sine wave component corresponding to those in the swept-sine-wave test is accurately sampled and processed, as a result of the well-designed test parameters. This is why the test accuracy can be maintained in such a rapid test. Therefore, the MFE method proposed in this paper can be used for rapid and accurate dynamic testing of micromachined gyroscope chips and in other similar electromechanical systems.

## 5. Conclusions

A novel MFE method has been proposed in this paper to replace the traditional SFE method for rapid and accurate dynamic testing of micromachined gyroscope chips. Using the proposed method, the processing time was reduced from 10 min to 6 s when testing one chip under four modes at a 1-Hz frequency resolution and 601-Hz bandwidth. The experimental results demonstrated that the MFE method can achieve the same repeatability and accuracy as the traditional SFE method. Thus, the proposed MFE method can potentially meet the urgent need for gyroscope chip filtering and pairing or packaged gyroscope calibration for large-scale gyroscope manufacturing. The MFE method can also be applied to the dynamic testing of other electronic and electromechanical systems with voltage-signal excitation.

## Figures and Tables

**Table 1:** Repeatability and accuracy test results using the sweep-frequency excitation (SFE) and multifrequency excitation (MFE) methods.

**Test Results of Gyroscope Chips**	**Sense Axis Input– Sense Axis Output Mode**		**Drive Axis Input– Drive Axis Output Mode**	

	**Mean**	**Standard Deviation**	**Mean**	**Standard Deviation**
Resonant frequency using SFE [Hz]	3164	0	2956	0
Resonant amplitude using SFE [dB]	16.244	0.002	−10.458	0.001
Resonant phase using SFE [°]	91.103	0.021	86.660	0.039
Resonant frequency using MFE [Hz]	3164	0	2957	0
Resonant amplitude using MFE [dB]	16.245	0.003	−10.000	0.005
Resonant phase using MFE [°]	91.110	0.023	81.897	0.171
